# Modeling heterogeneous responsiveness of intrinsic apoptosis pathway

**DOI:** 10.1186/1752-0509-7-65

**Published:** 2013-07-23

**Authors:** Hsu Kiang Ooi, Lan Ma

**Affiliations:** 1 Department of Bioengineering, The University of Texas at Dallas, 800 W. Campbell Rd, Richardson, TX 75080, USA

**Keywords:** Intrinsic apoptosis pathway, Stochastic model, Intrinsic noise, Extrinsic noise

## Abstract

**Background:**

Apoptosis is a cell suicide mechanism that enables multicellular organisms to maintain homeostasis and to eliminate individual cells that threaten the organism’s survival. Dependent on the type of stimulus, apoptosis can be propagated by extrinsic pathway or intrinsic pathway. The comprehensive understanding of the molecular mechanism of apoptotic signaling allows for development of mathematical models, aiming to elucidate dynamical and systems properties of apoptotic signaling networks. There have been extensive efforts in modeling deterministic apoptosis network accounting for average behavior of a population of cells. Cellular networks, however, are inherently stochastic and significant cell-to-cell variability in apoptosis response has been observed at single cell level.

**Results:**

To address the inevitable randomness in the intrinsic apoptosis mechanism, we develop a theoretical and computational modeling framework of intrinsic apoptosis pathway at single-cell level, accounting for both deterministic and stochastic behavior. Our deterministic model, adapted from the well-accepted Fussenegger model, shows that an additional positive feedback between the executioner caspase and the initiator caspase plays a fundamental role in yielding the desired property of bistability. We then examine the impact of intrinsic fluctuations of biochemical reactions, viewed as intrinsic noise, and natural variation of protein concentrations, viewed as extrinsic noise, on behavior of the intrinsic apoptosis network. Histograms of the steady-state output at varying input levels show that the intrinsic noise could elicit a wider region of bistability over that of the deterministic model. However, the system stochasticity due to intrinsic fluctuations, such as the noise of steady-state response and the randomness of response delay, shows that the intrinsic noise in general is insufficient to produce significant cell-to-cell variations at physiologically relevant level of molecular numbers. Furthermore, the extrinsic noise represented by random variations of two key apoptotic proteins, namely Cytochrome C and inhibitor of apoptosis proteins (IAP), is modeled separately or in combination with intrinsic noise. The resultant stochasticity in the timing of intrinsic apoptosis response shows that the fluctuating protein variations can induce cell-to-cell stochastic variability at a quantitative level agreeing with experiments. Finally, simulations illustrate that the mean abundance of fluctuating IAP protein is positively correlated with the degree of cellular stochasticity of the intrinsic apoptosis pathway.

**Conclusions:**

Our theoretical and computational study shows that the pronounced non-genetic heterogeneity in intrinsic apoptosis responses among individual cells plausibly arises from extrinsic rather than intrinsic origin of fluctuations. In addition, it predicts that the IAP protein could serve as a potential therapeutic target for suppression of the cell-to-cell variation in the intrinsic apoptosis responsiveness.

## Background

Apoptosis, the major form of programmed cell death, is a conserved cell suicide process critical for the health and survival of multicellular organisms [1-3]. Apoptosis plays a fundamental role in animal development, by sculpting tissues and structures, as well as in tissue homeostasis, by regulating and maintaining balanced cell number [4-6]. Dysregulation of apoptosis is associated with various human diseases, ranging from developmental disorders, neurodegeneration to cancer [7,8].

**Figure 1:**
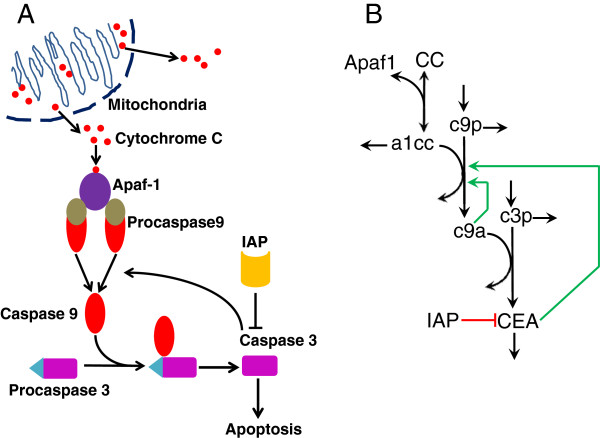
**Intrinsic apoptosis signaling pathway.(A)** Schematic diagram of the intrinsic or mitochondria apoptosis signaling pathway. Upon the release of Cytochrome C from mitochondria, it binds with apoptotic protease activating factor 1 (Apaf-1) to form the apoptosome. The apoptosome activates procaspase-9 into caspase 9, the initiator apoptotic caspase. Caspase 9 then cleaves and activates procaspase-3 to caspase3, the executioner or effector caspase. In turn, caspase 3 promotes the activation of caspase 9, forming a positive feedback loop. Meanwhile, the inhibitor of apoptosis protein (IAP) inhibits the activity of caspase 3. Elevated activity of caspase 3 induces irreversible fate of apoptosis. **(B)** Reaction scheme of our mathematical model of intrinsic apoptosis pathway, where Apaf-1, Cytochrome C, apoptosome, procaspase-9, caspase 9, procaspase-3 and caspase 3 are denoted by Apaf1, CC, a1cc, c9p, c9a, c3p and c3a, respectively. Note that the black arrows represent direct reactions such as binding, synthesis and degradation, the red line with hammerhead represents inhibiting regulatory reaction, and the two green arrows represent activating regulations, which are the modifications of the Fussenegger model.

Apoptosis is regulated by two interrelated signaling pathways: the extrinsic or death-receptor pathway, and the intrinsic or mitochondrial pathway [1,9]. They converge on the execution pathway, mediated intracellularly by a cascade of cysteine proteases, termed caspases [10,11]. Caspases are specialized cysteine proteases found in animal cells as inactive procaspases (proenzymes). Through proteolytic cleavage, procaspases are activated to carry out its apoptotic mission. The intrinsic pathway begins with the release of Cytochrome C (CC) from mitochondria through membrane permeabilization [12], triggered by intracellular stress such as DNA damage and hypoxia [9] (Figure 1A). Once CC translocates to the cytosol, it binds to apoptotic protease activating factor 1 (known as Apaf-1) to form a multimeric protein complex called the apoptosome. This apoptosome complex then activates the initiator procaspase, called procaspase-9. The activated caspase (caspase-9) cleaves the executioner procaspase (procaspase-3) to form active executioner/effector caspase (CEA), whereby the apoptotic response is irreversibly triggered [11]. Experiments have shown that the activation of effector caspases occurs in an all-or-none fashion, emphasizing the functional role of the apoptosis system as a molecular switch. In the past several years, advances in experimental skills have allowed the measurement of apoptosis dynamics in individual cells [3,13-16], confirming the switch-like dynamics, while revealing another feature of prominent stochasticity in the apoptotic responses at single-cell level.

Since the key constituents and molecular interactions of apoptosis pathways have been experimentally identified, the approach of mathematical modeling and computer simulations have been employed extensively to help elucidate the complicated regulatory network and dynamic responsiveness related to apoptosis at average cellular population level [14,15,17-19]. Nevertheless, recent experiments at single-cell resolution have discovered noisy phenotypic diversity of apoptosis activity in that significant cell-to-cell heterogeneity of the dynamic apoptosis responses exist across a genetically-identical cell population [16]. Toward understanding such single-cell variability in apoptosis response, some theoretical efforts have been taken recently to model the stochastic response of receptor-mediated apoptotic pathway. The stochastic behavior of intrinsic apoptosis pathway, on the other hand, has been the subject of relatively little mathematical modeling to date. In this work, we will focus on addressing the intrinsic apoptosis pathway under stochastic perturbations by developing theoretical and computational models at single-cell level. The models will be exploited to investigate the heterogeneous behavior of intrinsic apoptosis network among individual cells.

Deterministic model based on ordinary differential equations (ODEs) is the most widely used mathematical approach to describe the molecular kinetics during cell death signaling. Fussenegger et al. developed a well-accepted ODE model that integrates components of the extrinsic as well as the intrinsic apoptosis pathways [20]. Qualitatively the Fussenegger model compares reasonably well with published experimental kinetics of caspase activation at average cell population level. Nevertheless, there is lack of understanding of the nonlinear stability and systems properties of this model, which hinders deeper understanding of the system behavior. For instance, studies have suggested that bistability is a key system feature for apoptotic signaling networks [15,16,21-23], which could achieve the all-or-none responses and in addition confer robustness to the apoptosis system [18,24,25]. It is unclear whether the Fussenegger model presents the property of bistability. Since then, there have been considerable theoretical efforts on modeling and systems analysis using ODE models of death-receptor mediated apoptosis [17,18,26,27], mitochondria-mediated apoptosis [28,29], or integrated extrinsic and intrinsic apoptosis pathways [15,30-34].

The past few years have seen increasing efforts in stochastic modeling to address the heterogeneous apoptosis responses at single-cell level. Specifically, these efforts incorporate cellular noise perturbations into the apoptosis framework. Cellular noise is defined as stochastic fluctuations of biomolecular processes within and between cells. It can be divided into intrinsic noise and extrinsic noise [35,36]. Intrinsic noise in genetically identical cells refers to random deviation of the molecular processes from their average deterministic kinetics within a cell, mostly due to probabilistic biochemical reactions associated with low copy number of molecular quantities [35,37]. Extrinsic noise arises from global perturbation factors such as cellular environment and organelle distribution, which results in cell-to-cell variation in rate constants of biochemical reactions, expression levels of genes and proteins, and other parameters of biochemical processes [35,38,39]. Towards the analysis of cellular noise, several statistical measures of noise have been proposed to quantify the level of stochastic fluctuations of biomolecular processes [40-43]. Two measures of noise are commonly used to characterize the stationary averages and variances of random cellular components. In particular, noise strength can be quantified by Fano factor, which is defined as the steady-state variance over average and has a value of 1 for Poisson process. The Fano factor of an arbitrary stochastic system reveals deviations from Poissonian behavior [44-46]. A more standard and frequently used measure of cellular noise is the dimensionless coefficient of variation, which is defined as standard deviation divided by mean. It measures the inverse signal-to-noise ratio and has been widely employed to characterize intrinsic and extrinsic noises of gene and protein expression and their determining factors with respect to cellular network organization [36,43,47-52]. For the latter measure, the coefficient of variation squared may be alternatively used [53]. In this work, we use the coefficient of variation to quantify the noise of the random distribution of molecular components and stochastic cellular response time. In the aspect of mathematical modeling of noise in apoptosis pathway, several previous stochastic apoptosis models have taken into account of the intrinsic noise by either applying Gillespie’s stochastic simulation algorithm to the ODE models or constructing Monte Carlo models from first principles [22-24,54,55]. With regard to modeling the impact of extrinsic noise on apoptosis pathway, there have been a few studies notably only on receptor-mediated apoptosis pathway [16,56].

In this study, we attempt to develop mathematical and computational models of the intrinsic apoptosis pathway at single-cell level, and to identify plausible sources of non-genetic heterogeneity of apoptosis dynamics observed in a cell population using stochastic simulations. We start with a deterministic ODE model of intrinsic apoptosis pathway adapted from the Fussenegger model and find that bistability is missing. By adding positive feedback regulations that are supported by previous experimental evidences, we develop a model of intrinsic apoptosis pathway that functions as a bistable switch. We are particularly interested in understanding the stochastic behavior of this apoptosis switch under perturbation of intrinsic noise and/or extrinsic noise. Stochastic modeling and simulations of intrinsic apoptosis pathway indicate that noise could enhance robustness of the bistable switch. In addition, we show that intrinsic noise is not sufficient to induce the observed level of cell-to-cell variability of apoptosis response at biologically relevant level of molecular numbers, while the extrinsic noise of protein variations is plausibly the main source giving rise to the degree of heterogeneous responses of intrinsic apoptosis pathway between single cells.

## Results and discussion

### Deterministic model of intrinsic apoptosis pathway

To build a single-cell level deterministic model of intrinsic apoptosis pathway, we adopt the ODE modeling framework of intrinsic apoptosis proposed by Fussenegger et al. [20] as it has the “minimal” model complexity while preserving all the critical interactions of intrinsic apoptosis pathway. The model scheme is shown in Figure 1B and summarized here: the intrinsic apoptosis pathway is initiated by the release of Cytochrome C (CC) from mitochondria to cytosol, and binding to Apaf-1 to form the apoptosome complex (denoted as a1cc). The apoptosome catalyzes procaspase-9 (denoted as c9p), the precursor of initiator caspase, to its active form, caspase 9 (denoted as c9a). The executioner procaspase-3 (denoted as c3p) is then activated by c9a to form the active executioner caspase 3 (denoted as CEA), whose response represents the onset of the irreversible apoptosis fate. Finally, CEA is subject to the direct inhibition by IAP protein [20] (please refer to Table 1 for the description of all the abbreviation terms).

**Table 1:** Summary of abbreviation terms

**Abbreviation**	**Full name or description**
Apaf-1	Apoptotic protease activating factor 1
a1cc	Apoptosome complex formed by Cytochrome C and Apaf-1
CC	Cytochrome C
CEA	Executioner caspase 3
CV	Coefficient of variation
c3p	Executioner procaspase-3
c9a	Initiator caspase 9
c9p	Initiator procaspase-9
IAP	Inhibitor of apoptosis proteins
ODE	Ordinary differential equation
SSA	Stochastic simulation algorithm
*T*_*d*_	Caspase 3 response delay time

**Figure 2:**
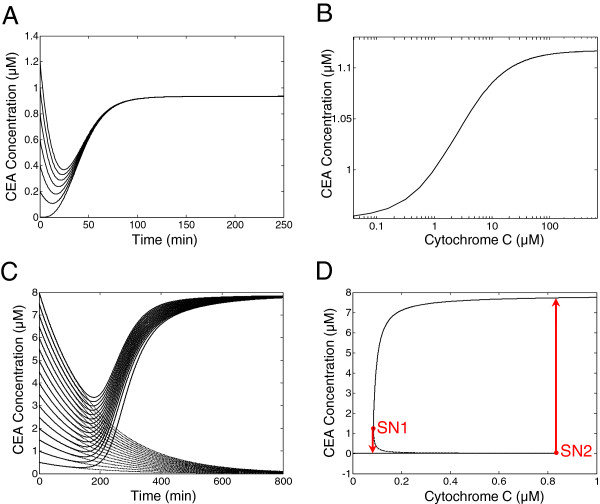
**Simulation and analysis of deterministic models of intrinsic apoptosis pathway.(A)** Simulations of the Fussenegger ODE model of intrinsic apoptosis pathway show that the time courses of caspase 3 (CEA) starting from various initial conditions all converge to single steady state. **(B)** Bifurcation analysis of the Fussenegger model. Plot of steady-state CEA versus input signal Cytochrome C (CC) shows that the model is monostable. **(C)** Simulations of our ODE model modified based on Fussenegger model show that the time courses of CEA can converge either to a high steady state, if the input signal is high (solid line), or to a near-zero steady state, if the input signal is low (dashed line). **(D)** Bifurcation analysis of our model confirms that CEA has two stable steady states (upper and lower branches of black solid lines) and an unstable steady state (middle branch of dashed line). The bifurcation diagram has two saddle-node (SN) bifurcation points at CC = 0.08 *μ*M (SN1) and CC = 0.83 *μ*M (SN2). CEA jumps from one sable steady state to the other stable steady state if CC shifts below SN1 or above SN2 as illustrated by the red arrows. Therefore, the modified apoptosis model is bistable, and the bistability domain between SN1 and SN2 allows the system to resist against mild input perturbation.

Simulations of this ODE model demonstrate that the time trajectories of CEA starting from an initially low concentration first undergo some time delay and then switch to a high steady state with a relatively sharp transition (Figure 2A). Nevertheless, the time trajectories of CEA from different initial conditions all converge to the same steady-state level, indicating the existence of single equilibrium point. Indeed, bifurcation analysis of this ODE model with respect to the parameter of CC concentration shows that the steady-state of CEA is monostable with a sigmoidal input-output relationship (Figure 2B). This means that regardless of different input strengths and different initial conditions, the response would asymptotically settle at only one steady state. Further analytical analysis of the steady-state response of the Fussenegger model proves that the mapping between the input of CC signal and the output of CEA is either one-to-one or one-to-two and thus there exist at most two solutions of the output signal at equilibrium (Additional file 1: Supporting Information). As a result, the Fussenegger model of intrinsic apoptosis pathway cannot be bistable as bistability of a system requires three distinct steady-state solutions in some range of input. The Fussenegger model of intrinsic apoptosis pathway presents a system property of so-called ultrasensitivity (or threshold response) rather than bistability [57,58]. This, however, is inconsistent with the current consensus of bistability feature of apoptosis system, and thus extra model refining step is needed to resolve the discrepancy. To this end, we modify the existing model by incorporating a positive feedback from the executioner caspase (CEA) to the initiator caspase (c9a), an auto-catalysis loop to the activation kinetic of c9a, and a mild cooperativity in CEA activation induced by c3p, which are supported by previous experimental and computational findings (Figure 1) [29,59-62]. The existing mathematical models accounting for intrinsic apoptosis pathway differ from our proposed model in choice of apoptosis pathway. Specifically, we follow the Fussenegger model to assume that the inhibition of caspase 9 by IAP is not essential and ignored in our model. This choice is the main distinction from the model proposed by Legewie *et al*[28], where IAP inhibits both caspase 9 and caspase 3 thus giving rise to an IAP-mediated positive feedback and bistability. The Harrington *et al* model [33], the Zhang et al model[29], and the Kutumova et al model [34] all inherit the schematic of the intrinsic apoptosis model proposed by Legewie et al, and thus also include the double inhibitions of caspase 9 and caspase 3 by IAP and the resulting implicit positive feedback.

**Figure 3:**
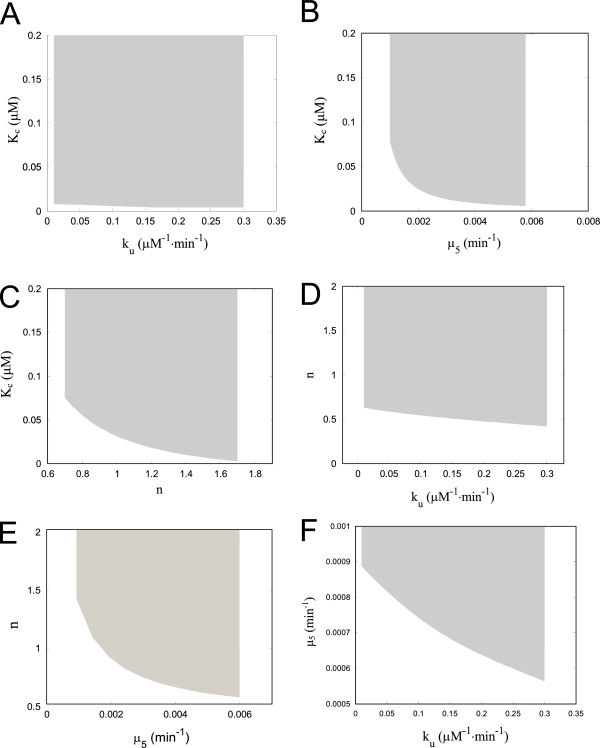
**Two-parameter bifurcation diagrams of the modified model of intrinsic apoptosis pathway.** Bifurcation analysis of our deterministic model against pairs among four selected parameters, namely Hill Constant in the positive feedback loop (*K*_*c*_), cooperativity in the activation of CEA (*n*), degradation rate of caspase 9 (*μ*_5_), and rate constant of the inhibition of CEA by IAP (*k*_*u*_). 2D bistability diagrams are plotted with respect to *K*_*c*_ and *k*_*u*_**(A)**, *K*_*c*_ and *μ*_5_**(B)**, *K*_*c*_ and *n***(C)**, *n* and *k*_*u*_**(D)**, *n* and *μ*_5_**(E)**, *μ*_5_ and *k*_*u*_**(F)**. The gray area depicts the location of paired parameter values at which the CEA response is bistable.

The governing ODEs of the modified model of intrinsic apoptosis pathway are listed in the Methods section. Simulations of the modified ODE model show that when given a step input of low concentration of CC, the time courses of CEA gradually settle at a near-zero steady state starting from different initial CEA concentrations, while given a relatively high concentration of CC, CEA eventually settles at a high steady state (Figure 2C). Such behavior with two stable output steady states indicates that bistability is achieved by the modified ODE model. In addition, the time trajectories agree with experimental results in that the CEA response is not elicited until after a few hours of delay time (>2hrs).The switching-on kinetic of CEA activation is sigmoidal shape and completed within ∼1hr, presenting all-or-none switch-like behavior [13,63]. Indeed, one-parameter bifurcation analysis of the modified ODE model confirms that the steady-state response of CEA is bistable with respect to the input signal of CC, where two stable steady states coexist between the input concentrations of 0.08 *μ*M and 0.83 *μ*M (Figure 2D). The bifurcation curve has two saddle-node bifurcation points SN1 and SN2, giving rise to a middle unstable branch and two stable branches, where the upper and lower branches correspond to the apoptosis and survival fates, respectively. When the concentration of CC approaches SN1 and SN2, hysteretic behavior occurs: the system remains at near-zero CEA activity at low amount of CC, until an ON threshold (0.83 *μ*M) is reached, whereby CEA activity switches to the apoptosis state abruptly; inversely, the CEA activity switches from the apoptosis state to the survival state only if the CC concentration falls below the OFF threshold (0.08 *μ*M). The system properties of bistability and hysteresis could confer robustness to the apoptotic responsiveness by allowing cells that are not committed to apoptosis to remain at survival state, even in the event of mildly noisy input. In addition, two-dimensional bifurcation analysis with respect to four selected parameters, namely the Hill constant that regulates the CEA-mediated positive feedback loop (*K*_*c*_), the cooperativity of activation of CEA (*n*), the degradation rate of c9a(*μ*_5_) and the inhibition rate of CEA by IAP (*k*_*u*_), show that the bistability property of the modified model exists in extended parameter space around the nominal parameter set (Figure 3) and is hence a robust behavior.

### Stochastic model of intrinsic apoptosis pathway under intrinsic noise perturbation

The deterministic model of the intrinsic apoptosis pathway has allowed us to analyze nonlinear properties of the system and quantify region of robust behavior. Nevertheless, the ODE modeling approach accounts for average cellular dynamics while ignoring the inevitable unpredictability embedded in biomolecular reactions and in intra- and extra-cellular environments. It has been observed by several different experimental techniques that the apoptosis response at single-cell resolution is subject to inherent stochastic perturbations, giving rise to pronounced cell-to-cell variability even in genetically identical cell population [13,14,16]. Therefore, it is necessary for us to develop a stochastic modeling framework of intrinsic apoptosis pathway, which can be used to explore the plausible origin of the stochasticity underlying the phenotypic heterogeneity.

**Figure 4:**
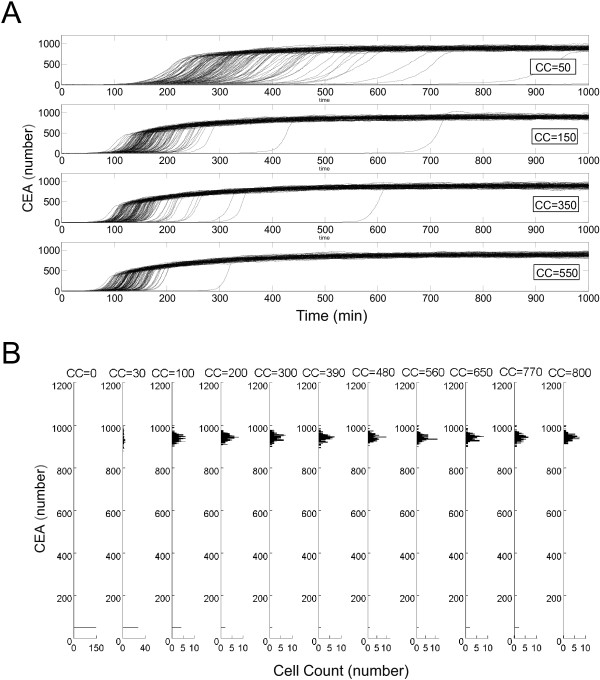
**Stochastic simulation of the model of intrinsic apoptosis pathway under intrinsic noise.(A)** Stochastic model under intrinsic noise is simulated using the modified Gillespie stochastic simulation algorithm as described in the Methods. Simulation results of 150 time trajectories of CEA with [c9p, c3p] =[ 10^4^,10], representing the stochastic response under intrinsic noise in 150 cells, are plotted at different number of CC molecules. It is evident that under intrinsic noise perturbation the time trajectories of CEA present cell-to-cell variability when the number of CC molecules is below 1000. **(B)** Histograms of the steady-state CEA values of the 150 cells simulated with [c9p, c3p] =[ 10^4^,10] and at varying input CC level. They exhibit bimodal distribution around two steady states, one at CEA = 960 (number of molecules) and the other at CEA = 55 (number of molecules). The bimodal distribution implies that the stochastic model under intrinsic noise is bistable.

In this study, we investigate the impact of both intrinsic-noise and extrinsic-noise perturbations on the intrinsic apoptosis dynamics. First, to model the stochasticity under the intrinsic-noise perturbation due to low copy number of biomolecules, we assume that the deterministic model represents the nominal single-cell behavior, and apply the standard Gillespie SSA (stochastic simulation algorithm) [64,65]. The detail of applying SSA to the deterministic model can be found in the Methods section. To reflect the plausible low molecular copy numbers arising in the intrinsic apoptosis pathway within single cells, we perform simulations under different combinations of number of molecules that fall into the experimentally observed ranges [66]. Furthermore, in choosing number of molecules used for stochastic simulation of intrinsic noise, we limit the number to be within [ 0,10^4^] in general, as previous experimental and computational work have suggested that the coefficient of variation of cellular signal due to intrinsic noise reaches a minimal level when the mean abundance of molecules increases to 10^4^[48,49]. Using this limit and previous experimental observation that procaspase-9 (c9p) and procaspase-3 (c3p) could have relatively low number of molecules per cell, ranging respectively from 5×10^3^ to 1.6×10^5^ and from undetectable to 1.6×10^6^[66], we choose to run SSA of the model at molecule numbers of c9p = {5×10^3^,10^4^} and c3p = {10,10^2^,10^3^,10^4^}, which correspond to a total of eight combined conditions. Note that each realization of stochastic simulation corresponds to the apoptosis response of one single cell. Figure 4A shows 150 stochastic time trajectories of output CEA response with [c9p, c3p] =[ 10^4^,10], representing the behavior of 150 cells, under varying amount of Cytochrome C input. Each time course of CEA activation exhibits sigmoidal switch-like shape, converging to an elevated steady state.

To illustrate the cellular variability in the stochastic response due to intrinsic noise, histograms of the steady-state CEA copy numbers in the same 150 cells is plotted in Figure 4B against different input CC levels. The histogram shows that if the CC molecule is above 15 copy number the distribution of steady-state CEA is bimodal, with a low mean steady-state value of ∼50 number of CEA molecules and a high mean steady-state value of ∼960 CEA molecules. Such bimodal distribution of CEA response indicates that the stochastic response of intrinsic apoptosis pathway subject to intrinsic noise under this particular c9p/c3p condition is bistable [67]. The bistability behavior persists even when the copy number of CC increases to ∼800 molecules, showing that the corresponding fold change of bistability domain under intrinsic noise is above five times that of the deterministic model, where the bistability region of CC is [0.08, 0.83] (*μ*M) as shown in Figure 2D. Such phenomenon of enhanced robustness induced by intrinsic noise is in agreement with previous computational work which suggests that stochastic signaling networks may perform more robustly than their deterministic counterpart [68,69]. It is noteworthy that the existence and range of bistable CEA response are dependent on the abundances of c9p and c3p in that the bistability boundary shrinks as the copy numbers of procaspases increase, and the bistable range becomes almost undetectable at significantly high amount of procaspases (10^4^) (Additional file 2: Table S1). This result implies that the bistability property of apoptosis might only be observable at proper condition of molecular abundance.

**Figure 5:**
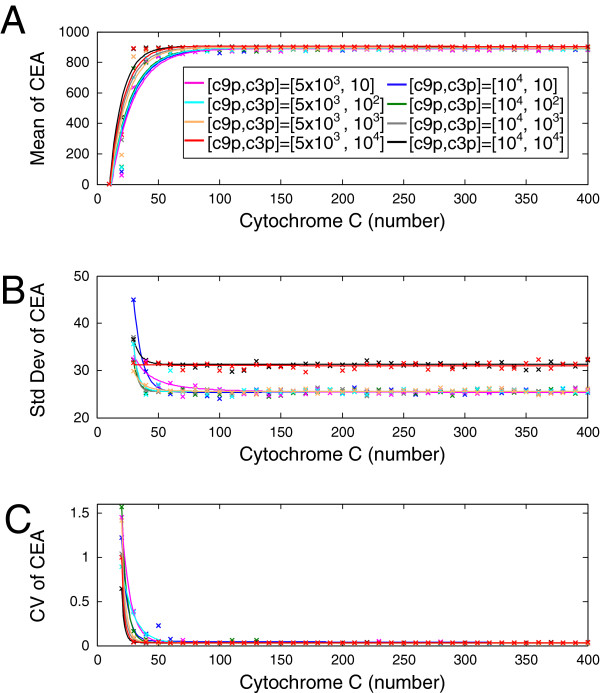
**Effect of intrinsic noise on the stochasticity of steady-state CEA response.** Stochastic model of intrinsic apoptosis pathway subject to intrinsic noise is simulated under the eight copy-number conditions of c9p = {5×10^3^,10^4^} combined with c3p = {10,10^2^,10^3^,10^4^}. **(A)** Mean level of the steady-state CEA response is plotted versus the input signal CC (cross: simulation data; solid line: fitted curve). Calculation is based on 150 cells at each data point. The color scheme of specific copy-number condition of [c9p, c3p] is as follows: magenta for [ 5×10^3^,10]; cyan for [ 5×10^3^,10^2^]; orange for [ 5×10^3^,10^3^]; red for [ 5×10^3^,10^4^]; blue for [ 10^4^,10]; green for [ 10^4^,10^2^]; gray for [ 10^4^,10^3^]; black for [ 10^4^,10^4^]. **(B)** Standard deviation of the steady-state CEA response is plotted versus the input signal CC (cross: simulation data; solid line: fitted curve). The same color scheme as **(A)** is used. **(C)** The coefficient of variation (CV) of the steady-state CEA response is plotted versus the input signal of CC (cross: simulation data; solid line: fitted curve). All the calculation is based on 150 cells at each data point. The same color scheme as **(A)** is used.

Since Figure 4B has demonstrated that the apoptosis state of CEA varies from cell to cell with a normal distribution, next we evaluate the degree of stochasticity of the CEA response at equilibrium caused by intrinsic noise. We use the coefficient of variation (CV) to quantify the level of stochasticity, following previous studies of isogenic cell-to-cell variability of apoptosis response [3,16,70]. As shown in Figure 5A and 5B, under all the eight conditions of molecule numbers as described above, the mean value of the stochastic CEA response increases while its standard deviation decreases as the input signal CC increases, both with exponential pattern. Note that the condition of higher c9p and c3p abundances give rise to elevated mean response of CEA at equilibrium as well as sharper stimulus-response curve, agreeing with the pro-apoptotic roles played by these two components. The consequent stochasticity of the output CEA value, quantified by CV, monotonically decreases with increasing amount of input and reaches a minimum level as the number of CC molecules is above 60 for all the eight conditions (Figure 5C). This indicates that the intrinsic noise in the steady-state behavior of intrinsic apoptosis response at single cell level can be suppressed by enhancing the strength of input signal. Note also that lower abundances of c9p and c3p induce more prominent stochasticity in output when the input amount of CC is sufficiently low.

**Figure 6:**
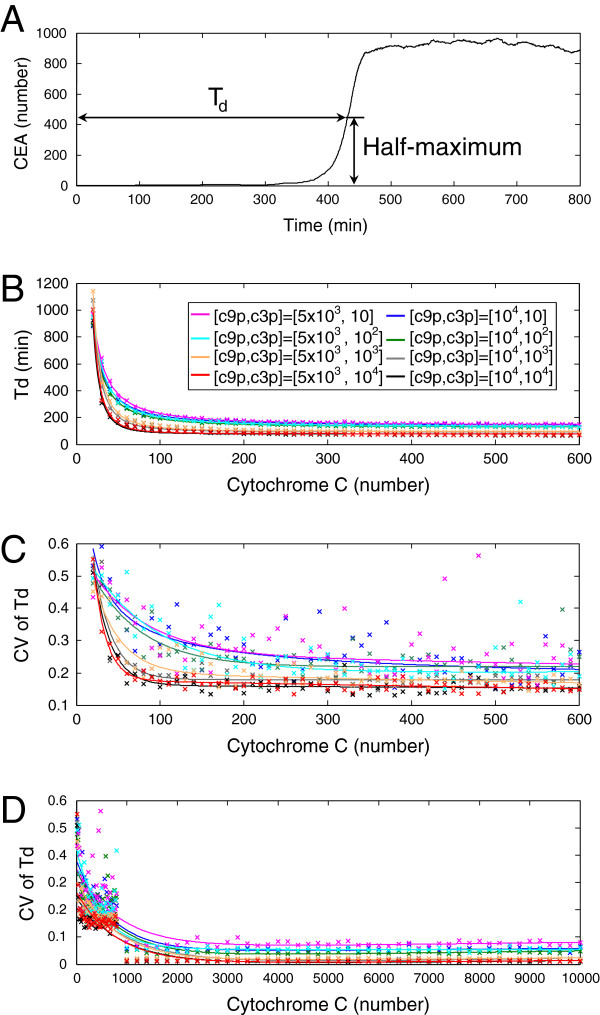
**Effect of intrinsic noise on the stochasticity of the time delay of CEA response.** Stochastic model of intrinsic apoptosis pathway subject to intrinsic noise is simulated under the eight copy-number conditions of c9p = {5×10^3^,10^4^} combined with c3p = {10,10^2^,10^3^,10^4^}. **(A)** The time delay of CEA activation, denoted as *T*_*d*_, is defined as the time it takes to reach half maximal of the mean steady-state value of CEA response. **(B)** The mean value of *T*_*d*_ is plotted versus the input signal CC (cross: simulation data; solid line: fitted curve). The color scheme of specific copy-number condition of [c9p, c3p] is as follows: magenta for [ 5×10^3^,10]; cyan for [ 5×10^3^,10^2^]; orange for [ 5×10^3^,10^3^]; red for [ 5×10^3^,10^4^]; blue for [ 10^4^,10]; green for [ 10^4^,10^2^]; gray for [ 10^4^,10^3^]; black for [ 10^4^,10^4^]. **(C)** The cell-to-cell stochasticity of *T*_*d*_ represented by the coefficient of variation of *T*_*d*_ is plotted versus the input signal CC in the range of 0 to 600 number of molecules (cross: simulation data; solid line: fitted curve). The same color scheme as **(B)** is used. **(D)** The coefficient of variation (CV) of *T*_*d*_ is plotted versus CC in the range of 0 to 10,000 number of molecules (cross: simulation data; solid line: fitted curve). The same color scheme as **(B)** is used. All the calculation is based on 150 cells at each data point.

We then focus on an important quantity that has been experimentally measured to evaluate the cell-to-cell variability in apoptosis response. As demonstrated in Figure 6A, each stochastic time trajectory of CEA presents a time delay between time zero, the CC release time, and the CEA activation time, which is conventionally defined as the half-maximal caspase 3 cleavage time. Such delay time (denoted by *T*_*d*_ hereafter) is defined analogously to the quantity previously used in experiments to characterize the response time of extrinsic apoptosis pathway in single cells [3,15,16]. And experimental quantifications suggest that *T*_*d*_ varies from cell to cell, even in genetically identical clones [3,16]. Our single-cell stochastic simulations with intrinsic noise, under the eight conditions of c9p and c3p abundances, show that in general the mean value of *T*_*d*_ monotonically decreases with increasing CC level (Figure 6B). For instance, when c9p and c3p are respectively 10^4^ and 10^2^ molecules per cell, the mean value of *T*_*d*_ decreases from around 900 min at 20 number of CC molecules to ∼160 min at 600 number of CC molecules. Understandably, lower abundances of pro-apoptotic proteins c9p and c3p lead to longer response delay time. We then quantify the degree of cell-to-cell stochasticity by CV of *T*_*d*_. Figure 6C shows that the CV of *T*_*d*_ due to intrinsic noise is, under all the conditions, a monotonically decreasing function with regard to the number of CC molecules. Such findings of attenuated cellular variability of response under the circumstance of elevated input signal are consistent with previous experimental and computational results on the stochastic apoptosis pathway in genetically identical cells as well as other cellular processes [16,22,36,51].

The above stochastic simulations show that the cell-to-cell variability of intrinsic apoptosis due to intrinsic noise is especially evident when the number of c3p is relatively small (below 1000), where the CV of *T*_*d*_ persist at the level of non-genetic noise of apoptosis observed by experiments (CV ∼[0.2, 0.3]) [3,16] (Figure 6C). Moreover, the abundances of c9p and c3p inversely regulate the cellular variability among the eight conditions. Specifically, the lowest c9p/c3p copy-number condition leads to a CV slightly above 0.3 while the highest c9p/c3p copy-number condition leads to a CV slightly below 0.15 (Figure 6C), suggesting that the wide copy-number range of procaspases, especially procaspase-3, under the sub-1000 condition is likely a source of cell-to-cell variability under the perturbation of intrinsic noise. Previously, there has been study of apoptosis pathway implying that the molecular numbers of participating biochemical species seem to reside in a regime much higher than 1,000 [16]. Therefore, our stochastic model with intrinsic noise is further explored under the various copy numbers of c9p and c3p with sufficiently strong CC input. As shown in Figure 6D, under all the eight conditions the CV of *T*_*d*_ drops dramatically when the number of CC molecules increases above 1000. In particular, when the copy numbers of c9p, c3p and CC molecules are all raised to 1000 and above, the CV of *T*_*d*_ tends to an almost negligible level of ∼0.01. Such low degree of CV of *T*_*d*_ at physiologically more plausible condition indicates that the perturbation by intrinsic noise alone seems insufficient to induce the observed degree of cell-to-cell stochasticity of apoptosis dynamics (that is, CV ∼[0.2, 0.3]), and other sources of uncertainty needs to be taken into account.

### Stochastic model of intrinsic apoptosis pathway under extrinsic noise perturbation

Recently, natural protein variations across cell population has been identified as a major source of extrinsic fluctuations for apoptosis pathway [16]. Experiments have suggested that the concentration of a protein naturally varies among different cells following a log-normal distribution with a typical CV value of 0.2 to 0.3 [16,71]. To address the impact of this kind of extrinsic noise on intrinsic apoptosis pathway, we assume that in the deterministic single-cell model of intrinsic apoptosis pathway described above certain protein(s) of interest has log-normally distributed concentration with CV equal to 0.25.

**Figure 7:**
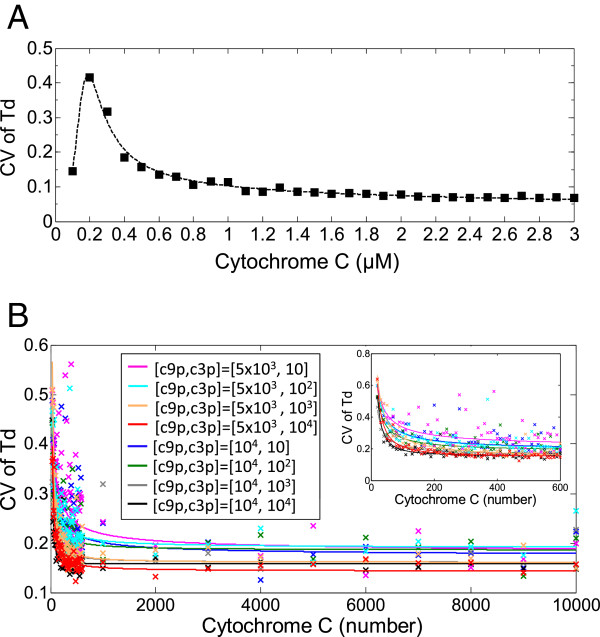
**Effect of extrinsic noise in the amount of Cytochrome C protein on the stochasticity of the time delay of CEA response.(A)** Stochastic model of intrinsic apoptosis pathway under the extrinsic noise of random amount of Cytochrome C (CC) is implemented by simulating the deterministic model, while assuming the mean concentration of CC is log-normally distributed with a coefficient of variation CV = 0.25 across cells. The coefficient of variation (CV) of the time delay of CEA activation (denoted as *T*_*d*_) is plotted versus the concentration of CC. **(B)** Stochastic model under both intrinsic noise and the extrinsic noise of random amount of Cytochrome C (CC) is simulated using Gillespie algorithm, while assuming the mean number of CC is log-normally distributed with a coefficient of variation CV = 0.25 across cells. Eight copy- number conditions of c9p = { 5×10^3^,10^4^} combined with c3p = { 10,10^2^,10^3^,10^4^} are implemented in the simulations. The coefficient of variation (CV) of *T*_*d*_ is plotted versus the number of CC molecules in the range of [0, 10000]. In the inset plot, the coefficient of variation (CV) of *T*_*d*_ is zoomed into the range of [0, 600] number of CC molecules. Cross: simulation data; solid line: fitted curve. The color scheme of specific copy-number condition of [c9p, c3p] is as follows: magenta for [ 5×10^3^,10]; cyan for [ 5×10^3^,10^2^]; orange for [ 5×10^3^,10^3^]; red for [ 5×10^3^,10^4^]; blue for [ 10^4^,10]; green for [ 10^4^,10^2^]; gray for [ 10^4^,10^3^]; black for [ 10^4^,10^4^]. All the calculation is based on 150 cells at each data point.

**Figure 8:**
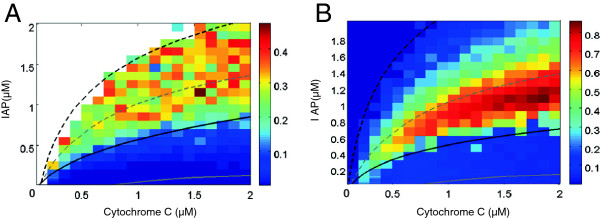
**Effect of extrinsic noise in the amount of IAP protein on the stochasticity of the time delay of CEA response.(A)** Stochastic model of intrinsic apoptosis pathway under the extrinsic noise of random amount of IAP protein is implemented by simulating the deterministic model, while assuming the mean concentration of IAP is log-normally distributed with a coefficient of variation CV = 0.25 across cells. Coefficient of variation (CV) of the time delay of CEA activation, *T*_*d*_, is shown as 2D heat map versus the mean concentration of IAP protein at various concentrations of Cytochrome C (CC). The superimposed curves are the low (dashed lines) and high (solid lines) thresholds of the bistability diagram of the corresponding stochastic model (black color) as well as the deterministic model (gray color). The blank region means zero CV of *T*_*d*_ as all the CEA trajectories have zero steady-state value. **(B)** Stochastic model under the extrinsic noise of random amount of both IAP and CC proteins is implemented by simulating the deterministic model, while assuming the concentrations of both CC and IAP are log-normally distributed with a coefficient of variation CV = 0.25 across cells. Coefficient of variation (CV) of the time delay of CEA activation, *T*_*d*_, is shown as 2D heat map versus the mean concentration of the IAP protein and that of the CC protein. Again, the superimposed curves are the low (dashed lines) and high (solid lines) thresholds of the bistability diagram of the corresponding stochastic model (black color) as well as the deterministic model (gray color).

First, we simulate the stochasticity due to variation in the concentration of the protein CC, the critical input signal of intrinsic apoptosis pathway, using the single-cell deterministic model with a randomly generated CC concentration. Again, as a measure of cell-to-cell variability, the CV of *T*_*d*_ among 150 cells is calculated at varying mean values of CC protein (Figure 7A). The variability peaks (CV =∼ 0.42) at mean concentration of CC = 0.2 *μ*M, and then declines as the mean abundance of CC protein increases, settling at a value of CV = ∼0.1 eventually. It indicates that the natural variation in the CC protein can induce some degree of cell-to-cell stochasticity to the intrinsic apoptosis response, especially at relatively low abundance of CC protein. Second, we evaluate the impact of extrinsic noise driven by the natural variation of IAP protein, which is one of the most pivotal anti-apoptotic proteins tightly regulating apoptosis via antagonizing the activity of CEA [72,73]. The single-cell deterministic model plus a log-normally distributed amount of IAP with CV equal to 0.25 is simulated for 150 cells at different concentrations of input CC protein. Here, we plot the resultant CV of *T*_*d*_ evaluated among 150 cells in Figure 8A as a 2-dimensional (2D) heat map, versus the concentration of CC protein and the mean concentration of IAP protein. It shows that given the same CC input, higher mean concentration of IAP, albeit with the same dispersion of random distribution, leads to relatively larger CV of *T*_*d*_ and hence higher degree of cell-to-cell variability in the timing of CEA activation. Most likely, large amount of IAP significantly represses the activity of CEA, while it is generally known that the lower amount of a biochemical component the higher degree of its stochasticity. Additionally, Figure 8A indicates that at the same mean abundance of stochastic IAP protein, higher input level of CC protein yields lower cell-to-cell variability in the apoptosis response. The above findings together imply that the extrinsic noise in CC protein has opposite influence on the induction of cell-to-cell variability to intrinsic apoptosis response than the extrinsic noise in IAP protein. Such contrary effect is in line with their opposite regulatory roles in apoptosis pathway, in that CC is pro-apoptotic while IAP is anti-apoptotic. Notably, Figure 8A also shows that if the mean concentration of IAP is moderately high (1−2*μ*M), the CV of *T*_*d*_ can achieve a value of 0.3 or even higher, which is the level of cell-to-cell stochasticity measured by experiments. Hence the extrinsic noise in moderate amount of IAP protein may serve as a significant source of cell-to-cell variability in intrinsic apoptosis response. But if IAP is so high that paramount of inhibition is produced, the apoptosis response of CEA is then constantly switched off, denoted by the upper-left non-responsive region in the 2D heat map (blank area in Figure 8A).

It is interesting to find out that when the 2D heat map is superimposed with the boundary of the two-parameter bistability diagram of the stochastic model, which is estimated using the histograms of CEA response (black curves in Figure 8A), the CV of *T*_*d*_ with relatively high values (0.2–0.45) is located between the low-threshold curve (broken black line) and the high-threshold curve (solid black line). Such behavior is confirmed under systematic perturbations of the parameter *K*_*c*_ (Additional file 3: Figure S2) and the other three parameters selected for the sensitivity analysis in Figure 3 (data not shown). Compared to the threshold lines for the bistability domain of the corresponding deterministic model (gray curves), the extrinsic noise in CC seems not significantly affect the area of bistability domain, albeit the thresholds of CC shift toward smaller values. This trend also holds under different values of *K*_*c*_ (Additional file 3: Figure S2).

As a further exploration of the impact of extrinsic noise, we allow the concentrations of both the CC and IAP proteins to be log-normally distributed random numbers with CV equal to 0.25. The resulting 2D heat map of the CV of *T*_*d*_ in Figure 8B shows that the level of stochasticity across cells is in general higher than the case under individual extrinsic perturbation of CC protein or IAP protein. In most of the area in the 2-parameter region of Figure 8B, the CV of *T*_*d*_ achieves a value above 0.2. Similar to Figure 8A, the CV of *T*_*d*_ with double extrinsic noises attains highest values (up to 0.8) between the low-threshold and high-threshold curves estimated for the stochastic bistability diagram, which is almost twice the CV value in the scenario under the extrinsic noise in IAP protein only. Moreover, the activation of CEA response can now be elicited even in the non-responsive region of Figure 8A due to the additional degree of fluctuation in the input signal. Collectively, the 2D heat-map in Figure 8B suggests that the extrinsic noises in CC and IAP proteins are sufficient to yield the experimentally observed degree of cell-to-cell variability in the apoptosis response.

The bistability domains under the nominal value of *K*_*c*_ in Figure 8B and those under systematically perturbed values of *K*_*c*_ in Additional file 4: Figure S3 demonstrate the same trend of behavior as those in Figure 8A and Additional file 3: Figure S2. Close comparison between Figure 8A/Additional file 3: Figure S2 and Figures 8B/Additional file 4: Figure S3 indicate that the area of bistability domain on average is slightly larger in the latter group. Therefore, additional degree of extrinsic noise may induce extra robustness for bistability of apoptosis.

### Stochastic model of intrinsic apoptosis pathway under combined intrinsic and extrinsic noise perturbations

We have so far analyzed the model of intrinsic apoptosis pathway subject to either intrinsic noise or extrinsic noise independently. Additionally, we would like to find out if both types of noises are present in the intrinsic apoptosis pathway, how the cell-to-cell variability of the delay time of CEA response is influenced. To simulate a model under the perturbation of combined intrinsic and extrinsic noises, we implement the stochastic simulation algorithm of the apoptosis model as described above to mimic the intrinsic noise, and simultaneously allow certain protein concentrations to be log-normally distributed random variables to represent the extrinsic noise. In order to make comparison with the results of the intrinsic-noise only case, we again use the eight conditions of molecule numbers at c9p = {5×10^3^,10^4^} in combination with c3p = { 10,10^2^,10^3^,10^4^}.

First, the impact of extrinsic fluctuation in the amount of CC protein on top of the intrinsic noise is simulated. The resulting CV of *T*_*d*_ in response to the abundance of CC less than 600 is shown in the inset of Figure 7B. Note that the unit of CC protein is now copy number of molecules rather than *μ*M as a requirement by the implementation of SSA. Comparing it to Figure 6C, we find that the behavior of cell-to-cell variability due to the combined types of noises is almost the same as that under intrinsic noise alone. That is, under all the eight molecule-number conditions, the CV of *T*_*d*_ monotonically decreases in a near exponentially-decaying fashion as the number of CC molecules increases, and it falls into similar range of value (∼[0.15, 0.3]). And similar to Figure 6C, the variability value is inversely correlated with the abundances of c9p/c3p, achieving CV of *T*_*d*_ above 0.2 if c9p/c3p have sub-1000 copy numbers. This result indicates that at the limit of low number of molecules (sub-1000), the intrinsic noise seems to make the dominating contribution to the cell-to-cell stochasticity of intrinsic apoptosis response.

We subsequently run the same stochastic model under the perturbation of intrinsic noise plus extrinsic noise in CC, while increasing the mean abundance of CC molecules up to 10,000, a more plausible reacting scale for apoptosis pathway. We find that the stochasticity curves under different c9p/c3p abundance conditions relatively converge at CC equal to 10,000 copy number (Figure 7B), due to diminished intrinsic noise. The comparison between Figure 7B and Figure 6D enables us to see the relative contributions of intrinsic and extrinsic noises: except for the case of almost undetectable abundance of c3p (= 10), which induce severe intrinsic noise, the CV of *T*_*d*_ due to combined noises is significantly higher than that due to intrinsic-only noise. Specifically, the contrast between the case of combined noises and the case of intrinsic-only noise is highest (CV ∼0.3 versus CV <0.02) when the copy numbers of c9p, c3p and CC molecules are all above 1000. Therefore, at higher molecular numbers, the cellular stochasticity seems to be majorly contributed by the source of extrinsic noise.

**Figure 9:**
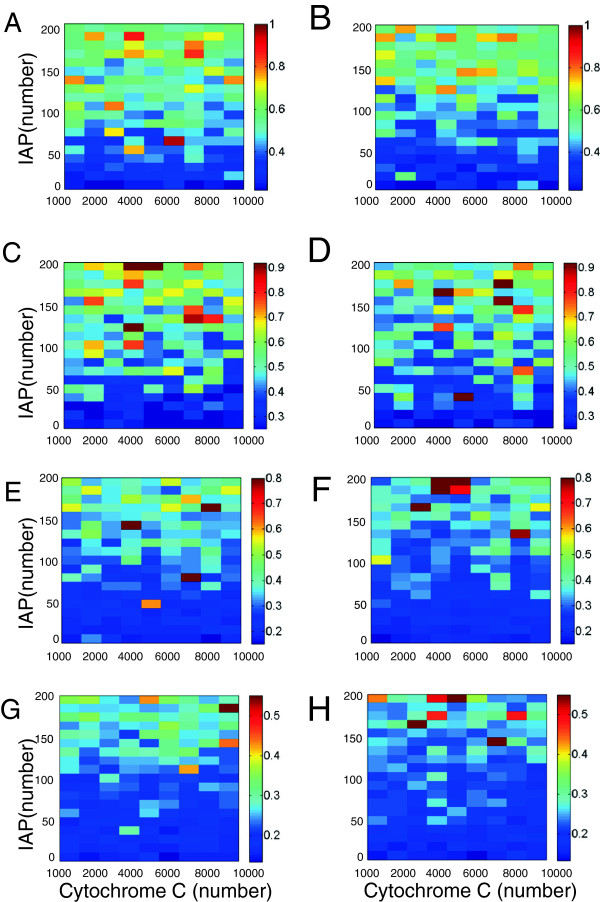
**Effect of combined intrinsic noise and extrinsic noise in the amount of Cytochrome C and IAP proteins on the stochasticity of the time delay of CEA response.** Stochastic model of intrinsic apoptosis pathway subject to intrinsic noise plus the extrinsic noise in the amount of Cytochrome C (CC) and IAP proteins is simulated using Gillespie algorithm, while assuming the mean numbers of CC and IAP proteins are log-normally distributed with a coefficient of variation CV = 0.25 across cells. Eight copy-number conditions of c9p = {5×10^3^,10^4^} combined with c3p = {10,10^2^,10^3^,10^4^} are implemented in the simulations. The resulting coefficient of variation of the time delay of CEA activation, *T*_*d*_, is plotted as 2D heat map versus the mean amount of CC and IAP proteins under specific copy-number condition of [c9p, c3p]: **(A)**, **(C)**, **(E)**, **(G)** respectively for [ 5×10^3^,10], [ 5×10^3^,10^2^], [ 5×10^3^,10^3^], [ 5×10^3^,10^4^], and **(B)**, **(D)**, **(F)**, **(H)** respectively for [ 10^4^,10], [ 10^4^,10^2^], [ 10^4^,10^3^], [ 10^4^,10^4^]. Note that the heat maps in each row use the same color scale for the convenience of comparison.

With respect to the possible impact of the extrinsic noise in IAP, we simulate the stochastic model with additional perturbation in the amount of IAP protein. That is, both the abundances of IAP and CC proteins are assumed to be log-normally distributed around their respective mean value with a CV of 0.25, alongside with intrinsic noise (again under the eight copy-number conditions). At the limit of low CC abundance the additional extrinsic noise of IAP does not introduce extra cell-to-cell stochasticity to apoptosis response (data not shown). When the mean number of CC molecules is in the physiologically plausible range of 1,000 to 10,000, simulations show that *T*_*d*_ presents a high CV, mostly above the value of 0.2 and even going up to a value of 1 in the 2D heat map (Figure 9). We compare the cell-to-cell variability under combined noises (Figure 9) with that under extrinsic-only noise (Figure 8B), and find that the CV of *T*_*d*_ increases only slightly in the former case (minimum value from ∼0 to ∼0.2, and maximum value from ∼0.85 to ∼1.05). This is consistent with the minor role of intrinsic noise played at elevated abundances of molecules. As a consequence, the boosted degree of stochasticity in Figure 9 has to owe predominantly to the extrinsic fluctuations in the amount of CC and IAP proteins rather than the intrinsic noise.

Simulations under the eight conditions of c9p and c3p abundances demonstrate that the overall cell-to-cell variability is, the same as what we see earlier, inversely correlated with the copy numbers of molecules, where the CV of *T*_*d*_ on average declines through row 1 to row 4 as the abundance of c3p increases from 10 to 10^4^, while the CV of *T*_*d*_ on average slightly declines from column 1 to column 2 as the abundance of c9p increases from 5×10^3^ to 10^4^. This is consistent with the aforementioned observation of the evident variability induced by the procaspase-3 under sub-1000 copy-number condition through the channel of intrinsic noise.

It is noteworthy that the CV of *T*_*d*_ in Figure 9 is generally larger at higher mean value of IAP molecular number, agreeing with the inference from Figure 8A that the abundance of IAP protein is capable of promoting the stochasticity of apoptosis pathway. It highlights the interesting role of IAP protein in controlling the cell-to-cell variability of intrinsic apoptosis response, and suggests that in treating diseases exploiting the apoptosis mechanism, such as cancer, the IAP protein offers a potential therapeutic target not only for effective modulation of apoptosis [74] but also for eliminating the undesired cell-to-cell heterogeneity, a major obstacle to effective cancer treatment and personalized medicine [75]. This finding is in line with the current view of the therapeutic function of IAP based on the study of apoptosis pathway at cell population level [17,76].

## Conclusions

The recently observed heterogeneous apoptosis phenotypes at single cell level have drawn increasing attention from researchers. Mathematical modeling and computer simulation provide an efficient approach to gain deep insight into the dynamic behavior of apoptosis network. This paper develops a theoretical and computational framework for single-cell stochastic modeling of the intrinsic apoptosis pathway. Using this modeling framework we explore the stochastic behavior of the intrinsic apoptosis response at single-cell level and seek to understand the plausible sources underlying the experimentally observed cell-to-cell variability of apoptosis response. We show that in the presence of noise, the bistable response of intrinsic apoptosis pathway can be more robust than its deterministic behavior. The coefficient of variation (CV) of the delayed timing of the activity of executioner caspase is utilized to quantify the stochasticity in the apoptosis dynamics. We find that the intrinsic noise can introduce significant cell-to-cell variability if the abundances of reacting biomolecules are relatively low. The level of cellular stochasticity solely due to intrinsic noise decreases dramatically to a negligible level of CV equal to ∼0.01 when the copy number of Cytochrome C is raised to 10,000, which is the amount suggested by a previous study. In addition, the extrinsic noise caused by the natural variations in protein concentrations of two key components in the intrinsic apoptosis pathway, Cytochrome C and the inhibitor of apoptosis (IAP) proteins, is also accounted for without or with intrinsic noise. Series of simulations indicate that the extrinsic noise is plausibly the major source of the cell-to-cell variability of intrinsic apoptosis response at high number of biomolecules. Furthermore, we find that the mean abundance of fluctuating IAP is positively correlated with the degree of cell-to-cell variability, thus making IAP a potential target for therapeutically suppressing the stochasticity of intrinsic apoptosis response across cell population in treating diseases such as cancer. In summary, this study based on our theoretical and computational models characterizes the behavior of the intrinsic apoptosis pathway under complex stochastic perturbations, and suggests that certain deterministic features, such as system bistability and IAP as potential therapy target, still remain in the presence of noise. Altogether, the work can enable us to gain deeper understanding toward the experimentally observed uncertainty in cellular decision making.

## Methods

### Deterministic ODE model

A deterministic model accounting for the average dynamics of intrinsic apoptosis pathway at single-cell level is developed. The model is adapted and modified based on the previous Fussenegger model [20]. Its reaction diagram and our modification method are given in the Results and discussion section. The model is formulated by 5-dimensional interconnected ODEs. The biochemical processes underlying each of the five ODEs are explained in detail as follows. Note that all the binding and unbinding processes are compactly represented by Michaelis-Menten kinetics under the quasi-steady-state assumption as previously described [20], and thus are not explained separately below.

Equation 1 accounts for the reversible association and disassociation of Cytochrome C (CC) with apoptotic protease-activator protein-1 (Apaf-1), assumed to be constant amount and lumped into parameter *k*_*f*1_, to form the Apoptosome complex (a1cc), and the degradation of apoptosome with a rate constant of *μ*_1_.

(1) $$\begin{array}{ll} \frac{d[\;a1\text{cc}]}{\text{dt}} & =\frac{k_{f1}[\;\text{CC}]}{1+K_{H}[\;\text{CC}]}-k_{r1}[\;a1\text{cc}]-\mu_{1}[\;a1\text{cc}]\; \end{array}$$

Equation 2 accounts for the synthesis of pro-caspase-9 (c9p) with a basal rate of Ω_9_, and the catalysis of c9p into caspase-9 (c9a) through binding with the apoptosome for activation, which is further promoted by executioner caspase 3(CEA) under positive feedback regulation, represented in the format of a first order Hill function, and also by c9a itself due to auto-catalysis. In addition, c9p is degraded with a rate constant of *μ*_2_.

(2) $$\begin{array}{ll} \begin{array}{cc} \;\frac{d[\;c9p]}{\text{dt}} & =\Omega_{9}-\frac{k_{f2}[\;\text{CEA}]}{[\;\text{CEA}]+K_{c}}\cdot \frac{[\;c9a]\cdot [\;a1\text{cc}]\cdot {\left[\;c9p\right]}^{2}}{\frac{1}{K_{K}\cdot K_{L}}\;+\;\frac{\left[c9p\right]}{K_{L}}+{\left[\;c9p\right]}^{2}}-\mu_{2}[\;c9p] \end{array} & \; \end{array}$$

Equation 3 accounts for the catalysis of c9a, which is the same as the process described by the second term of equation 2, and the degradation of c9a with a rate constant of *μ*_3_.

(3) $$\begin{array}{ll} \begin{array}{cc} \frac{d[\;c9a]}{\text{dt}} & =\frac{k_{f2}[\;\text{CEA}]}{[\;\text{CEA}]+K_{c}}\cdot \frac{[\;c9a]\cdot [\;a1\text{cc}]\cdot {\left[\;c9p\right]}^{2}}{\frac{1}{K_{K}\cdot K_{L}}+\frac{\left[c9p\right]}{K_{L}}+{\left[\;c9p\right]}^{2}}-\mu_{3}[\;c9a] \end{array} & \; \end{array}$$

Equation 4 accounts for the synthesis of the executioner pro-caspase-3 (c3p) at a basal rate of Ω_*EZ*_, activation into CEA through c3p binding with c9a and subsequent cleavage by c9a, where a cooperativity n=1.5 is included [29], and the degradation of c3p with a rate constant of *μ*_4_.

(4) $$\begin{array}{ll} \frac{d[\;c3p]}{\text{dt}} & =\Omega_{\text{EZ}}-k_{f3}\cdot {\left[\;c9a\right]}^{n}\cdot \frac{\left[\;c3p\right]}{\frac{1}{K_{P}}+c3p}-\mu_{4}[\;c3p]\; \end{array}$$

Equation 5 accounts for the activation of CEA, which is the same as the process described by the second term of equation 4, the inhibition of CEA by binding with IAP, represented by Michaelis-Menten kinetics, and the degradation of CEA with a rate constant of *μ*_5_. The amount of IAP is assumed to be constant and considered as a parameter of the model.

(5) $$\begin{array}{lll} \frac{d[\;\text{CEA}]}{\text{dt}} & =k_{f3}\cdot {\left[\;c9a\right]}^{n}\cdot \frac{\left[\;c3p\right]}{\frac{1}{K_{P}}+[\;c3p]}-\frac{k_{u}\cdot \text{IAP}\cdot [\;\text{CEA}]}{1+\text{IAP}\cdot K_{U}}\; & \;\\ & \;-\mu_{5}\cdot [\;\text{CEA}]\; \end{array}$$

The parameter values are mostly adopted from the Fussenegger model and listed in Table 2. The governing ODEs are solved by MATLAB ODE solver and the implementation can be found in Additional file 5: Model Script 1. Further bifurcation analysis is performed using the XPP-Auto freeware [77].

**Table 2:** **Nominal parameter values of the deterministic model, which are proposed based on the Fussenegger ***et al* model [20]

**Deterministic model parameter values**			
**Parameter**	**Description**	**Unit**	**Value**
*k*_*f*1_	Binding rate constant for Cytochrome C and Apaf-1	*min*^−1^	0.12
*k*_*f*2_	Catalysis rate constant for c9a	*μM*^−1^·*min*^−1^	0.3
*k*_*f*3_	Catalysis rate constant for CEA	*μM*^−1^·*min*^−1^	0.015
*k*_*r*1_	Unbinding rate constant for Cytochrome C and Apaf-1	*min*^−1^	0.4
*K*_*H*_	Equilibrium rate constant for binding of Cytochrome C and Apaf-1	*μM*^−1^	1
*K*_*K*_	Equilibrium constant for binding of c9p and Apoptosome	*μM*^−1^	0.5
*K*_*L*_	Equilibrium constant for binding of c9p and Apoptosome	*μM*^−1^	0.3
*K*_*P*_	Equilibrium constant for binding of c9a and c3p	*μM*^−1^	0.1
*K*_*U*_	Equilibrium constant for binding of IAP and CEA	*μM*^−1^	0.1
*k*_*u*_	Inhibition rate constant of CEA by IAP	*μM*^−1^·*min*^−1^	0.03
*IAP*	Concentration of free IAP protein	*μM*	0.018
*K*_*c*_	Threshold concentration for catalysis of c9p by CEA	*μM*	0.1
*μ*_1_	Degradation rate constant for the complex of Cytochrome C and Apaf-1	*min*^−1^	0.005
*μ*_2_	Degradation rate constant for c9p	*min*^−1^	0.005
*μ*_3_	Degradation rate constant for c9a	*min*^−1^	0.005
*μ*_4_	Degradation rate constant for c3p	*min*^−1^	0.005
*μ*_5_	Degradation rate constant for CEA	*min*^−1^	0.005
Ω_*EZ*_	Basal synthesis rate for c3p	*μM*·*min*^−1^	0.05
Ω_9_	Basal synthesis rate for c9p	*μM*·*min*^−1^	0.05

### Stochastic model with intrinsic noise

When the reacting species of a cellular signaling network have low molecular numbers, the inherent fluctuations of biochemical reactions become prominent. As a consequence, the deterministic formulation is no longer accurate to account for such effect of intrinsic noise, while probabilistic kinetic model is necessary. Toward this end, we refer to the Gillespie stochastic simulation algorithm (SSA) for simulating the stochastic biochemical kinetics with intrinsic noise. It is known that the standard SSA accounts for exact stochasticity of each molecule and every reaction event. To be applied to our ODE model, it requires expanding each term of the Michaelis-Menten kinetics into corresponding elementary reactions, which will add to significant computational demand especially when we intend to simulate more than one hundred single cells. Various recent studies have shown that using the lumped Hill functions for Michaelis-Menten kinetics in SSA actually yielded similar results as using the fully decomposed elementary biomolecular reactions model, thus validating the approach of applying quasi-steady-state assumption to reduce the complexity of stochastic models [78-81]. This modified Gillespie SSA is employed here, where the Michaelis-Menten kinetics is not expanded. Specifically, each binding reactions implemented by the Michaelis Menten kinetics in ODE is now treated as single reaction step, and the corresponding Hill function is incorporated directly as propensity function.

To implement the modified SSA, we decompose the deterministic model into 12 reaction elements according to the 12 biochemical reactions in the model, whose propensity functions are listed in Table 3. In particular, reaction elements 1, 2 and 3 refer to the reactions associated with apoptosome; reaction elements 4, 5 and 6 refer to the reactions associated with c9p; reaction elements 5 and 7 refer to the reactions associated with c9a; reaction elements 8, 9 and 10 refer to reactions associated with c3p; and lastly reaction elements 9, 11 and 12 refer to reactions associated with CEA. Then the standard SSA is implemented to simulate stochastic model in each cell, where each reaction is assigned with a reaction-occurrence probability and a random time interval for the next reaction, both dependent on its propensity function [64,65]. The algorithm updates the numbers of molecules for each reacting species and the probability of each reaction at every iteration. Each run of stochastic simulation represents response in a single cell. In order to compare behavior across a cell population, we choose a sample size of 150 cells, considering that previous experiments have used 100 single cells for statistical analysis of apoptotic response [16,82]. The above algorithm is written in MATLAB program (Additional file 6: Model Script 2).

**Table 3:** Elementary reactions of the stochastic model denoted by their corresponding propensity functions (PF)

**Rn**	**Elementary reaction (PF)**	**Rn**	**Elementary reaction (PF)**
Rn 1	$\frac{k_{f1}[\text{CC}]}{1+K_{H}[\text{CC}]}$	Rn 7	*μ*_**3**_[ *c*9*a*]
Rn 2	*k*_*r*1_[ *a*1*cc*]	Rn 8	Ω_*EZ*_
Rn 3	*μ*_**1**_[ *a*1*cc*]	Rn 9	$k_{f3}\cdot {\left[\;c9a\right]}^{n}\cdot \frac{\left[c3p\right]}{\frac{1}{K_{P}}+[c3p]}$
Rn 4	Ω_**9**_	Rn 10	*μ*_**4**_[ *c*3*p*]
Rn 5	$\frac{k_{f2}[\text{CEA}]}{[\text{CEA}]+K_{c}}\cdot \frac{[c9a]\cdot [a1\text{cc}]\cdot {\left[c9p\right]}^{2}}{\frac{1}{K_{K}\cdot K_{L}}+\frac{\left[c9p\right]}{K_{L}}+{\left[c9p\right]}^{2}}$	Rn 11	*μ*_**5**_[ *CEA*]
Rn 6	*μ*_**2**_[ *c*9*p*]	Rn 12	$\frac{k_{u}\cdot \text{IAP}\cdot [\text{CEA}]}{1+\text{IAP}\cdot K_{U}}$

### Stochastic model with extrinsic noise

As described in the Results and discussion section, the natural variation of protein concentration from cell to cell is considered as extrinsic noise in the apoptosis reactions. Our model of intrinsic apoptosis pathway subject to extrinsic noises in the Cytochrome C and IAP proteins is established using the above deterministic ODE model as the average single-cell model, with randomly selected parameter values as the varying protein concentrations. For instance, in each run of simulation the concentration of CC can be assumed to be a random number generated based on a log-normal distribution around its mean concentration with a CV of 0.25. Again, 150 independent runs of the stochastic model are generated to represent a sample size of 150 cells. To simulate the responses at different levels of CC signal, different mean concentrations of CC are used. Similarly, we can generate and simulate the stochastic model of intrinsic apoptosis pathway with extrinsic noise only in IAP protein, or with extrinsic noises in both CC and IAP proteins. The above algorithm is written in MATLAB program (Additional file 7: Model Script 3).

### Stochastic model with combined intrinsic and extrinsic noise

To establish a stochastic model with sources of both intrinsic and extrinsic noises, we employ the stochastic model with intrinsic noise, implemented by modified Gillespie SSA method as described above, while generating random abundances of CC protein and/or IAP protein. Again, the random protein is assumed to be log-normally distributed with a CV of 0.25. The simulation of stochastic models with intrinsic noise plus the extrinsic noises in CC and/or IAP proteins across a cell population is performed by a sample size of 150 cells. The above algorithm is written in MATLAB program (Additional file 8: Model Script 4).

The MATLAB codes for stochastic models are distributed to a high-performance computer cluster consisting of one master node and 96 slave nodes to achieve parallel computation that simulates responses in multiple single cells simultaneously.

## Competing interests

Both authors declare that they have no competing interests.

## Authors’ contributions

LM and HO developed the model. HO and LM performed simulations and analyses of the model. LM and HO drafted the manuscript. Both authors read and approved the final manuscript.

## Supplementary Material

Additional file 1 Supporting Information. Click here for file

Additional file 2 Table S1. Click here for file

Additional file 3 Figure S2. Click here for file

Additional file 4 Figure S3. Click here for file

Additional file 5 Model Script 1. Click here for file

Additional file 6 Model Script 2. Click here for file

Additional file 7 Model Script 3. Click here for file

Additional file 8 Model Script 4. Click here for file
