# Supplementary material for: Modeling heterogeneous responsiveness of intrinsic apoptosis pathway
Source: BMC Syst Biol. 2013 Jul 23;7:65. doi: 10.1186/1752-0509-7-65 (PMC3733900; doi:10.1186/1752-0509-7-65)
Supplement: Additional file 1 — Supporting Information. [file 1752-0509-7-65-S1.pdf]

Supporting Information

Steady-state solution of the Fussenegger model of intrinsic apoptosis pathway

A deterministic ODE model accounting for the intrinsic apoptosis signaling pathway has been proposed by Fussenegger et al. The 5-dimensional differential equations are listed below and the details of the model can be found in the main text:

$$\frac{d[\text{a1cc}]}{dt}=\frac{k_{f1}[\text{CC}]}{1+K_{\text{H}}[\text{CC}]}-k_{r1}[\text{a1cc}]-\mu_1[\text{a1cc}] \quad \text{(S1)}$$

$$\frac{d[\text{c9p}]}{dt}=\Omega_9-k_{f2}\frac{[\text{a1cc}]\cdot[\text{c9p}]^2}{\frac{1}{K_K\cdot K_L}+\frac{[\text{c9p}]}{K_L}+[\text{c9p}]^2}-\mu_2[\text{c9p}] \quad \text{(S2)}$$

$$\frac{d[\text{c9a}]}{dt}=k_{f2}\frac{[\text{a1cc}]\cdot[\text{c9p}]^2}{\frac{1}{K_K\cdot K_L}+\frac{[\text{c9p}]}{K_L}+[\text{c9p}]^2}-\mu_3[\text{c9a}] \quad \text{(S3)}$$

$$\frac{d[\text{c3p}]}{dt}=\Omega_{\text{EZ}}-k_{f3}[\text{c3a}]\frac{[\text{c3p}]}{\frac{1}{K_P}+[\text{c3p}]}-\mu_4[\text{c3p}] \quad \text{(S4)}$$

$$\frac{d[\text{CEA}]}{dt}=k_{f3}[\text{c3a}]\frac{[\text{c3p}]}{\frac{1}{K_P}+[\text{c3p}]}-\frac{k_u\cdot\text{IAP}\cdot[\text{CEA}]}{1+\text{IAP}\cdot K_U}-\mu_5[\text{CEA}] \quad \text{(S5)}$$

In the following, we demonstrate analytically that this model cannot be bistable in caspase response. Specifically, we seek to find out when given a input signal of Cytochrome C whether the model can yield three steady-state solutions of the output caspase-3 (CEA) or not. Now assuming that the system equilibrium is reached and the left-hand-side of the equations (S1) through (S5) are set to be zero, we analyze the mapping between the input value [CC] and the steady-state output [CEA] by checking the steady-state solution of each of the five variables, namely [a1cc], [c9p], [c9a], [c3p] and [CEA], in five steps:

(1) The solution of (S1) with d[a1cc]/dt=0 is $[\text{a1cc}]_{\text{ss}}=\frac{k_{f1}}{k_{r1}+\mu_1}\frac{[\text{CC}]}{1+K_{\text{H}}[\text{CC}]}$. Therefore, given an input [CC] the solution of [a1cc] at steady state is unique and single.

(2) The equation (S2) with d[c9p]/dt=0 can be rearranged into a 3rd-degree polynomial with respect to the steady-state solution $[\text{c9p}]_{\text{ss}}$: $A[c9p]_{ss}^3+B[c9p]_{ss}^2+C[c9p]_{ss}+D=0$, where the polynomial coefficients are: $A=\mu_2/\Omega_9$, $B=k_{f2}/\Omega_9[a1cc]_{ss}+\mu_2/(\Omega_9K_L)-1$, $C=\mu_2/(\Omega_9K_KK_L)-1/K_L$ and $D=-1/(K_LK_L)$. For typical parameter values of apoptosis pathway, the polynomial coefficients satisfy: A>0, C≤0 (typically $\mu_2\le\Omega_9K_K$ [1]), and D<0. Under such constraints, it can be shown, by plotting the polynomial function $f=Ax^3+Bx^2+Cx+D$ and locating the region of its turning points (by finding the roots of df/dx=0), that the x-coordinate of the turning point concaving

downward is negative and the x-coordinate of the turning point concaving upward is positive. Therefore there exist only one or two non-negative solutions of $[c9p]_{ss}$, whose values are dependent on $[a1cc]_{ss}$.

(3) Setting d[c9p]/dt=0 and d[c9a]/dt=0, and substituting equation (S3) into equation (S2), we obtain a single unique solution: $[c9a]_{ss} = (\Omega_9 - \mu_2[c9p]_{ss}) / \mu_3$.

(4) Let d[c3p]/dt=0 and rearrange (S4), we obtain a quadratic equation with respect to $[c3p]_{ss}$: $\widetilde{A}[c3p]_{ss}^2 + \widetilde{B}[c3p]_{ss} + \widetilde{C} = 0$, where $\widetilde{A} = \mu_4$, $\widetilde{B} = k_{f3}[c9a]_{ss} + \mu_6 / K_p - \Omega_{EZ}$ and $\widetilde{C} = -\Omega_{EZ} / K_P$. With $\widetilde{A} > 0$ and $\widetilde{C} < 0$, it is straightforward to show that, regardless of the value of $\widetilde{B}$, for a quadratic equations there exists a single non-negative solution of $[c3p]_{ss}$ given $[c9a]_{ss}$.

(5) Finally, let d[c3p]/dt=0 and d[CEA]/dt=0, and substitute equation (S5) into equation (S4), we obtain a single unique solution: $[CEA]_{ss} = \dfrac{\Omega_{EZ} - \mu_4[\mathrm{c3p}]_{\mathrm{ss}}}{\mu_5 + \dfrac{k_u \cdot \mathrm{IAP}}{1 + \mathrm{IAP} \cdot K_U}}$.

In the above procedures, we have decomposed the pathway represented by the Fussenegger model into five sequential input-output relationships. Each input-output relationship of the five propagating steps has a one-to-one mapping except for the second step, which has a one-to-one or one-to-two mapping. Therefore, the Fussenegger model could only have one or two steady-state solutions of the output [CEA] given any input value of [CC]. Since a bistable system requires that there exists three steady-state solutions of the output in certain range of input signal, we can conclude that the Fussenegger model is not bistable.

Steady-state solution of our model of intrinsic apoptosis pathway with mutants of procaspase-9

In the following we determine the contribution of the two feedback mechanisms, namely the autocatalysis regulation of caspase 9 and the caspase 3-mediated activation of caspaspe 9 (the green arrows in Figure 1B), to the bistability of our model of intrinsic apoptosis pathway. Toward this end, we abolish these two feedback loops separately or simultaneously, mimicking three mutants of procaspase-9. Experimental evidences have suggested that the processing sites Asp-315 and Asp-330 of procaspase-9 are responsible for the autocatalysis regulation of caspase 9 and the caspase 3-mediated activation of caspaspe 9, respectively [2, 3]. We can therefore study the model response using the single mutant D315A, which is resistant to the autocatalysis of caspase 9, the single mutant D330, which is refractory to the caspase 3-mediated feedback activation of caspaspe 9, and the double mutant D315A/D330A, which have both feedback mechanisms blocked [3, 4]. Note that Stennicke et al have shown that the mutant

D315A/D330A still maintained partial activity comparing to the wild type case. To account for the partial activation, we assume that the mutation at Asp315 abolishes only the positive autoregulation of caspase 9, by removing the term [c9a] in equations (2) and (3) listed in the main text, while the feed-forward pathway of caspase 9 promoted by Apaf-1 still remains.

First, the two single-mutant models of D315A and D330A are simulated and the steady-state input-output relationships demonstrate that certain degree of bistability in the model is retained under both conditions (Figure S1). The residual bistability in both cases indicates that both of the positive feedback mechanisms contribute to the final bistable response of our complete model of intrinsic apoptosis pathway. The simulated steady-state response of the double mutant D315A/D330A, however, is not bistable indicating that removal of these two positive feedback loops abolish bistability (data not shown). This finding regarding the double mutant can also be confirmed analytically, using the same approach as the above analysis of the Fussenegger model, that the steady-state response of the D315A/D330A model is indeed not bistable, as the only difference in the proof is to replace $[c9a]_{ss}$ by $[c9a]_{ss}^{n}$ in step 4 and the final conclusion remains the same.

As a summary, the study of the procaspase-9 mutants suggests that the two positive feedback loops embedded in our model are fully responsible for the bistability of the apoptosis response.

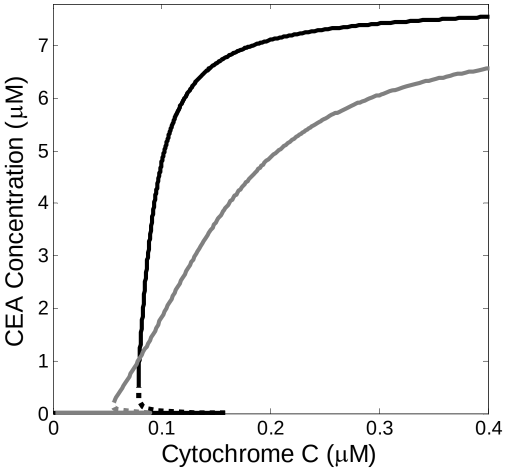

Figure S1. Bistability property persists for the model of the mutant D330A (black curve), where the feedback of caspase-3-mediated activation of caspase 9 is eliminated, as well as for the model of the mutant D315A (gray curve), where the autocatalysis of caspase 9 is removed. The solid lines represent stable solutions of CEA, while the dashed lines represent unstable solutions of CEA.

REFERENCES

1. Fussenegger M, Bailey JE, Varner J: **A mathematical model of caspase function in apoptosis**. *Nat Biotechnol* 2000, **18**(7):768-774.

2. Srinivasula SM, Ahmad M, Fernandes-Alnemri T, Alnemri ES: **Autoactivation of procaspase-9 by Apaf-1-mediated oligomerization**. *Mol Cell* 1998, **1**(7):949-957.
3. Zou H, Yang R, Hao J, Wang J, Sun C, Fesik SW, Wu JC, Tomaselli KJ, Armstrong RC: **Regulation of the Apaf-1/caspase-9 apoptosome by caspase-3 and XIAP**. *J Biol Chem* 2003, **278**(10):8091-8098.
4. Stennicke HR, Deveraux QL, Humke EW, Reed JC, Dixit VM, Salvesen GS: **Caspase-9 can be activated without proteolytic processing**. *J Biol Chem* 1999, **274**(13):8359-8362.
