# Supplementary material for: Modeling heterogeneous responsiveness of intrinsic apoptosis pathway
Source: BMC Syst Biol. 2013 Jul 23;7:65. doi: 10.1186/1752-0509-7-65 (PMC3733900; doi:10.1186/1752-0509-7-65)
Supplement: Additional file 2 — Table S1. [file 1752-0509-7-65-S2.pdf]

Table S1: Bistability boundary under different c9p/c3p copy-number conditions for the stochastic model with intrinsic noise.

The stochastic model of intrinsic apoptosis pathway subject to intrinsic noise is simulated at the following eight conditions of c9p/c3p molecular copy numbers. Listed are the resulting low and high thresholds of input Cytochrome C (CC), within which bistable responses of CEA exist.

**Number of c9p/c3p molecules**	**Low threshold**	**High threshold**
[c9p, c3p]= [$5x10^3$, 10]	CC=13	CC=780
[c9p, c3p]= [$5x10^3$, 10^2]	CC=12	CC=730
[c9p, c3p]= [$5x10^3$, 10^3]	CC=11	CC=70
[c9p, c3p]= [$5x10^3$, 10^4]	CC=10	CC=50
[c9p, c3p]= [10^4, 10]	CC=13	CC=770
[c9p, c3p]= [10^4, 10^2]	CC=11	CC=710
[c9p, c3p]= [10^4, 10^3]	CC=10	CC=35
[c9p, c3p]= [10^4, 10^4]	CC=10	CC=30
