# Supplementary material for: Modeling heterogeneous responsiveness of intrinsic apoptosis pathway
Source: BMC Syst Biol. 2013 Jul 23;7:65. doi: 10.1186/1752-0509-7-65 (PMC3733900; doi:10.1186/1752-0509-7-65)
Supplement: Additional file 3 — Figure S2. [file 1752-0509-7-65-S3.pdf]

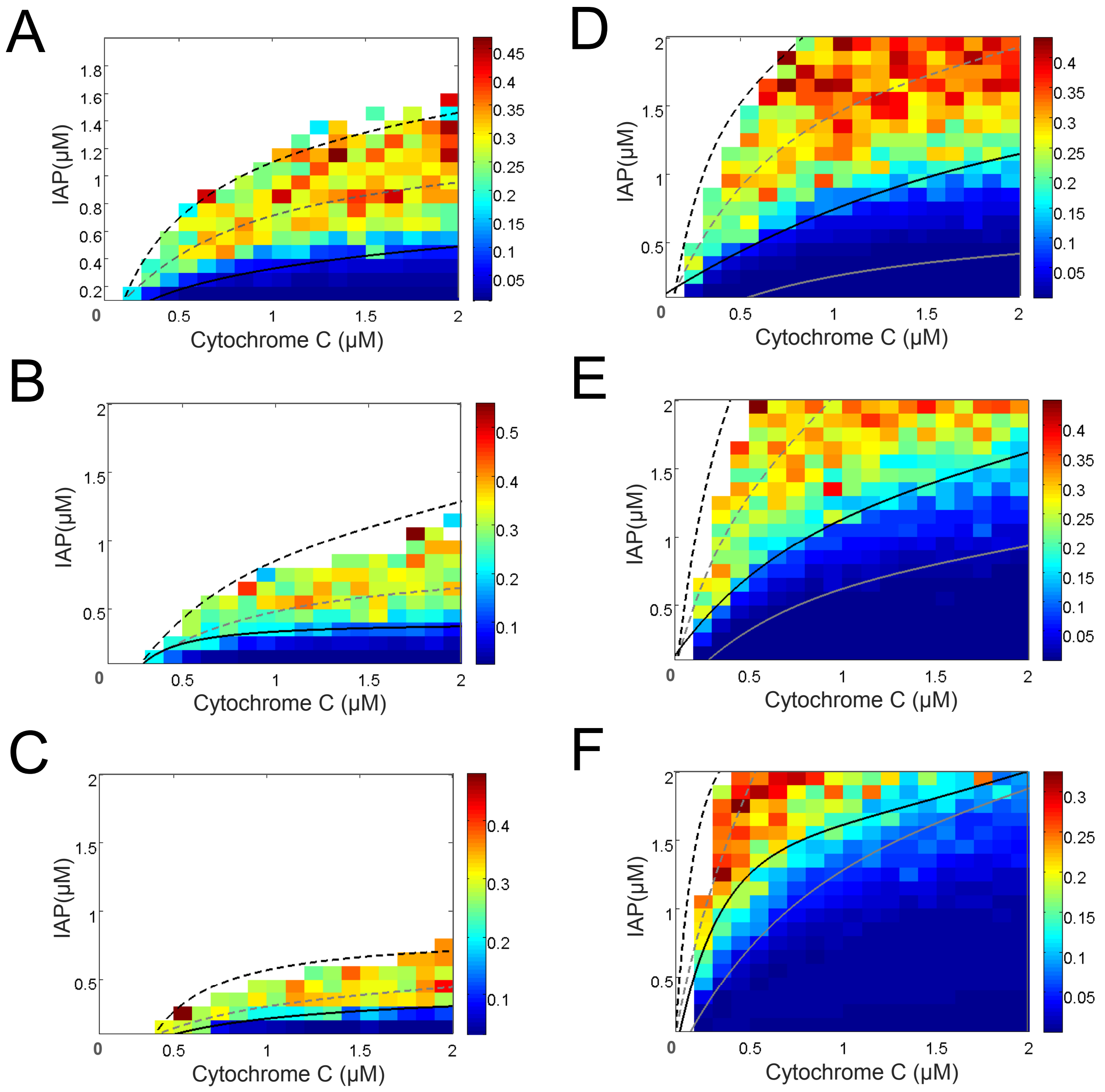

Figure S2. Sensitivity analysis of the result in Figure 8A by perturbation of the parameter K_c. The 2D heat map of the coefficient of variation of the time delay T_d is shown for stochastic model under the extrinsic noise of IAP, where K_c is 2x (A), 4x (B), 8x (C), 1/2x (D), 1/4x (E), and 1/8x (F) its nominal value. The curves superimposed are the low (dashed lines) and high (solid lines) thresholds of the bistability diagrams of the corresponding stochastic model (black color) and deterministic model (grey color). Note that the domain within which the CEA output displays bistable response (between the black dashed and solid lines) achieves the broadest area when K_c is half of its nominal value (D), and the bistable response domain rotates clockwise as the value of K_c increases.
