# Supplementary material for: Modeling heterogeneous responsiveness of intrinsic apoptosis pathway
Source: BMC Syst Biol. 2013 Jul 23;7:65. doi: 10.1186/1752-0509-7-65 (PMC3733900; doi:10.1186/1752-0509-7-65)
Supplement: Additional file 4 — Figure S3. [file 1752-0509-7-65-S4.pdf]

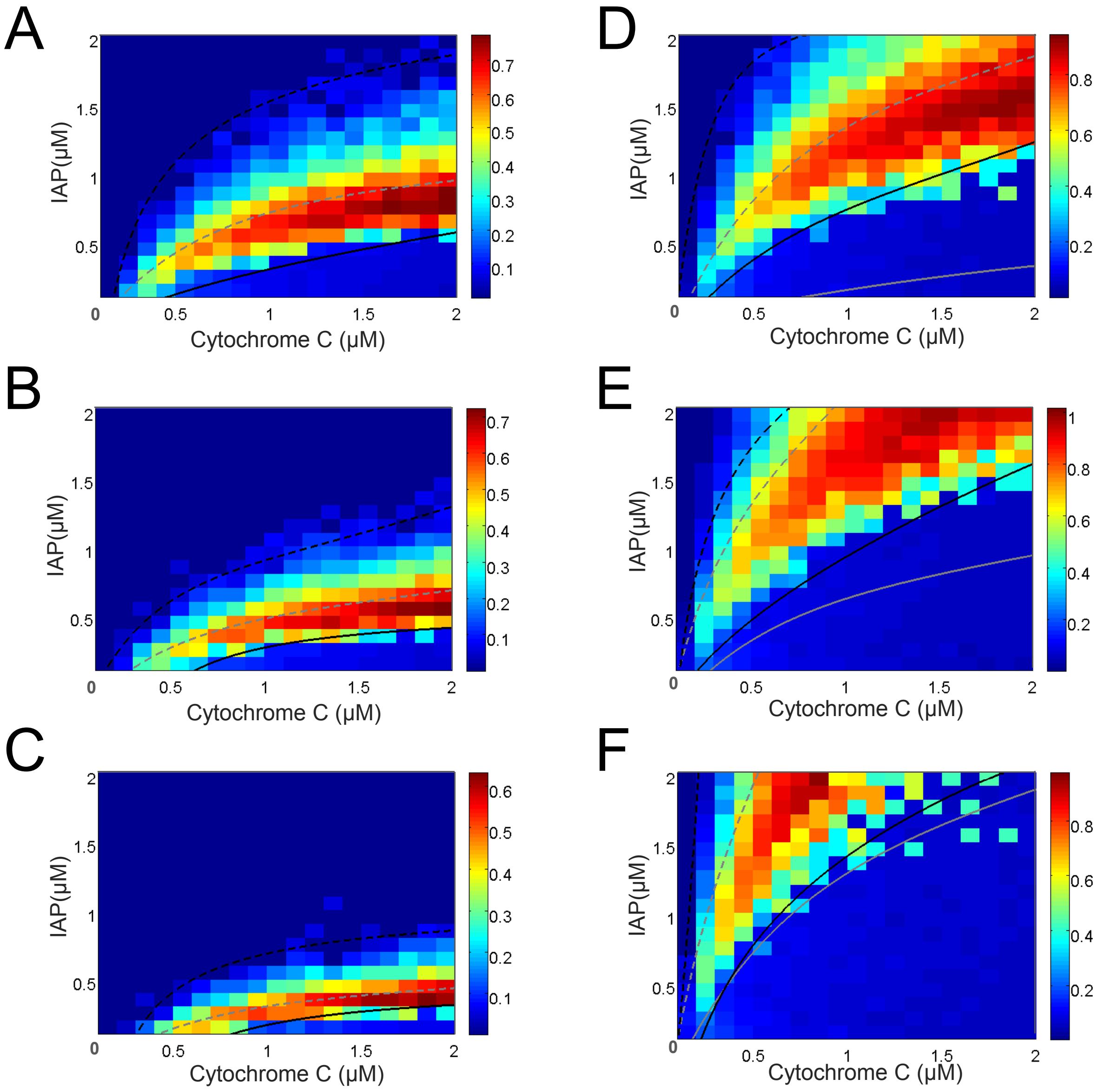

Figure S3. Sensitivity analysis of the result in Figure 8B by perturbation of the parameter K_c. The 2D heat map of the coefficient of variation of the time delay T_d is shown for stochastic model under the extrinsic noises of both IAP and Cytochrome C, where K_c is 2x (A), 4x (B), 8x (C), 1/2x (D), 1/4x (E), and 1/8x (F) its nominal value. The curves superimposed are the low thresholds (dashed lines) and high thresholds (solid lines) of the bistability diagrams of the corresponding stochastic model (black color) and deterministic model (grey color). Note that the domain within which the stochastic CEA output displays bistable response (between the black dashed and solid lines) achieves the broadest area when K_c is half of its nominal value (D), and the bistable response domain rotates clockwise as the value of K_c increases.
